# Expanding HPV Vaccine Among Young Adults: A Preventive Step Against Gynecological Cancers

**DOI:** 10.3390/vaccines14100916

**Published:** 2026-10-21

**Authors:** Seçil Topaloğlu Eti, İffet Güler Kaya, Aycan Bal, Rümeysa Ayhan, Melisa Uzunoğlu

**Affiliations:** School of Health Sciences, Istanbul Medipol University, İstanbul 34672, Turkey

**Keywords:** HPV, vaccine, young adult, cervical cancer, awareness, preventive health

## Abstract

Background: Human papillomavirus (HPV) is a major preventable cause of cervical and other HPV-related cancers. Despite the proven effectiveness of HPV vaccination, knowledge gaps and misconceptions continue to limit vaccine uptake among young adults. This study aimed to evaluate HPV awareness, attitudes and beliefs regarding HPV testing, and vaccination-related health beliefs among young adults and to examine the relationships among these constructs. Methods: This descriptive cross-sectional study was conducted with 313 young adults aged 18–25 years in Turkey. Data were collected through face-to-face interviews using a Personal Information Form, the HPV Awareness and Concern Scale, the HPV Testing Attitudes and Beliefs Scale, and the Health Belief Model Scale for HPV Infection and Vaccination. Data were analysed using descriptive statistics, independent-samples *t*-tests, one-way analysis of variance (ANOVA), Pearson correlation analyses, and post hoc tests, with statistical significance set at *p* < 0.05. Results: Although most participants were aware of HPV and its association with cervical cancer, substantial knowledge gaps remained regarding the appropriate timing and purpose of HPV vaccination. Most participants recognized that sexual activity was not a prerequisite for vaccination, whereas awareness of HPV-related cancers other than cervical cancer remained limited. HPV awareness was positively correlated with attitudes and beliefs regarding HPV testing (r = 0.239, *p* < 0.01) and vaccination-related health beliefs (r = 0.337, *p* < 0.01). Similarly, HPV testing attitudes and beliefs were positively associated with vaccination-related health beliefs (r = 0.228, *p* < 0.01). These findings indicate that, despite relatively high HPV awareness, important misconceptions and knowledge gaps regarding HPV vaccination persist among young adults. Conclusions: Targeted educational interventions, evidence-based social media campaigns, and healthcare professional-led counselling strategies may contribute to improving vaccine acceptance and strengthening HPV-related cancer prevention efforts.

## 1. Introduction

Cancer remains a major global public health concern and is associated with substantial morbidity, mortality, and socioeconomic burden. According to the World Health Organization, cancer is characterized by the uncontrolled growth of abnormal cells that may invade surrounding tissues and spread to other organs [1]. Among cancers affecting women, gynaecological malignancies constitute a significant public health challenge because of their effects on reproductive health, quality of life, and survival [2,3,4,5]. Cervical cancer is particularly important because it is largely preventable through effective vaccination, appropriate screening, and early treatment of precancerous lesions. Therefore, increasing awareness of cervical cancer risk factors and promoting participation in preventive healthcare services are essential components of cancer control strategies [3,4,6].

Human papillomavirus (HPV) infection is the primary etiological factor in cervical cancer development and is among the most common sexually transmitted infections worldwide [7,8]. Although most HPV infections resolve spontaneously, persistent infection with high-risk HPV types may lead to precancerous changes and cervical cancer. HPV infection is also associated with several other malignancies, including vulvar, vaginal, anal, penile, and oropharyngeal cancers. Prevention of HPV-related cancers involves complementary primary and secondary prevention strategies. HPV vaccination provides effective primary prevention against infections caused by vaccine-targeted HPV types, whereas cervical screening facilitates the early identification and management of precancerous lesions [7,9,10]. HPV DNA testing and cervical cytology are important screening approaches, with HPV DNA testing recommended as the preferred primary screening method in current international guidelines. Nevertheless, the effectiveness of these preventive strategies depends substantially on individuals’ knowledge, awareness, and willingness to engage in recommended health behaviours [6,9,11].

HPV vaccination is recognized as an important public health intervention for reducing the burden of HPV-associated diseases. Despite its established effectiveness, vaccination acceptance and uptake may be influenced by inadequate knowledge, misconceptions regarding vaccine safety, perceived susceptibility to infection, financial barriers, and limited access to reliable health information [9,11,12,13,14]. In Turkey, the implementation and accessibility of HPV vaccination have been important subjects of public health discussion. Previous studies conducted among university students and women in Turkey have identified variations in HPV-related knowledge, attitudes, and health beliefs, highlighting the importance of educational interventions and awareness-raising initiatives [9,10,12,13,14]. Young adulthood is a particularly relevant period for examining these factors because individuals increasingly make independent healthcare decisions and develop preventive health behaviours that may influence their long-term health outcomes.

Understanding HPV-related preventive behaviours requires consideration of not only knowledge but also the psychological and cognitive factors underlying health-related decision-making. The Health Belief Model, developed by Irwin M. Rosenstock and colleagues and subsequently elaborated by Rosenstock [15], provides a theoretical framework for explaining preventive health behaviours through individuals’ perceptions of susceptibility to disease, perceived severity of its consequences, perceived benefits of preventive actions, perceived barriers, and motivation to engage in health-protective behaviours. Within the context of HPV prevention, individuals who recognize their susceptibility to HPV infection and perceive vaccination as beneficial may demonstrate more favourable attitudes toward vaccination. Conversely, concerns regarding vaccine safety, cost, and perceived necessity may discourage vaccination-related decisions [7,10,14]. Similarly, attitudes and beliefs regarding HPV testing may reflect individuals’ understanding of HPV-related risks and their broader orientation toward preventive healthcare. Examining these interrelated constructs may provide a more comprehensive understanding of the factors associated with HPV vaccination decisions among young adults.

Although previous studies have investigated HPV knowledge, vaccination attitudes, and health beliefs in different populations, further research is needed to clarify the relationships among HPV awareness, HPV testing attitudes and beliefs, and HPV vaccination-related health beliefs among young adults in Turkey. Accordingly, this study aimed to evaluate HPV awareness, HPV testing attitudes and beliefs, and HPV vaccination-related health beliefs among young adults aged 18–25 years and to examine the relationships among these constructs. The study hypothesized that higher levels of HPV awareness and more favourable attitudes and beliefs toward HPV testing would be significantly associated with more favourable HPV vaccination-related health beliefs. By identifying these relationships, the findings may contribute to the development of targeted educational strategies and evidence-based interventions designed to strengthen HPV prevention awareness and support informed vaccination decisions.

## 2. Materials and Methods

### 2.1. Study Design, Participants, and Data Collection

This descriptive, cross-sectional study was conducted at a private hospital in Turkey to evaluate awareness, attitudes, and health beliefs regarding the human papillomavirus (HPV) among young adults. The study population consisted of individuals aged 18–25. The required sample size was estimated using the JotForm sample size calculator, based on a 95% confidence level and a 5% margin of error, determining a minimum sample size of 300 participants (The sample size was calculated using the online Sample Size Calculator provided by Jotform (Jotform Inc., San Francisco, CA, USA). The calculation was performed using the relevant study parameters to determine the minimum required sample size). Simple random sampling was used to select eligible participants [16]. Ultimately, 313 young adults who met the inclusion criteria were enrolled in the study. A total of 313 young adults—62% female and 38% male—participated in the study.

The inclusion criteria were being between 18 and 25 years of age, being able to read and understand Turkish, having the cognitive ability to respond to the research questions, and voluntarily agreeing to participate. Individuals with hearing, speech, or cognitive impairments that could prevent effective communication, those with a self-reported chronic illness, and those who declined participation or withdrew from the study were excluded.

Data were collected using a researcher-developed Personal Information Form and three validated measurement instruments assessing HPV-related awareness, attitudes, and health beliefs. The HPV Awareness and Concern Level Scale [17] demonstrated an overall reliability coefficient of 0.895, with subscale coefficients ranging from 0.863 to 0.882. The Attitudes and Beliefs About HPV Testing Scale [18] had an overall reliability coefficient of 0.875, with subscale coefficients ranging from 0.842 to 0.866. The Health Belief Model Scale Regarding HPV Infection and Vaccination [19] demonstrated an overall reliability coefficient of 0.912, with subscale coefficients ranging from 0.885 to 0.903. The internal consistency of the measurement instruments was also evaluated in the present study using Cronbach’s alpha coefficients, which were found to be within acceptable ranges.

**Figure 1:**
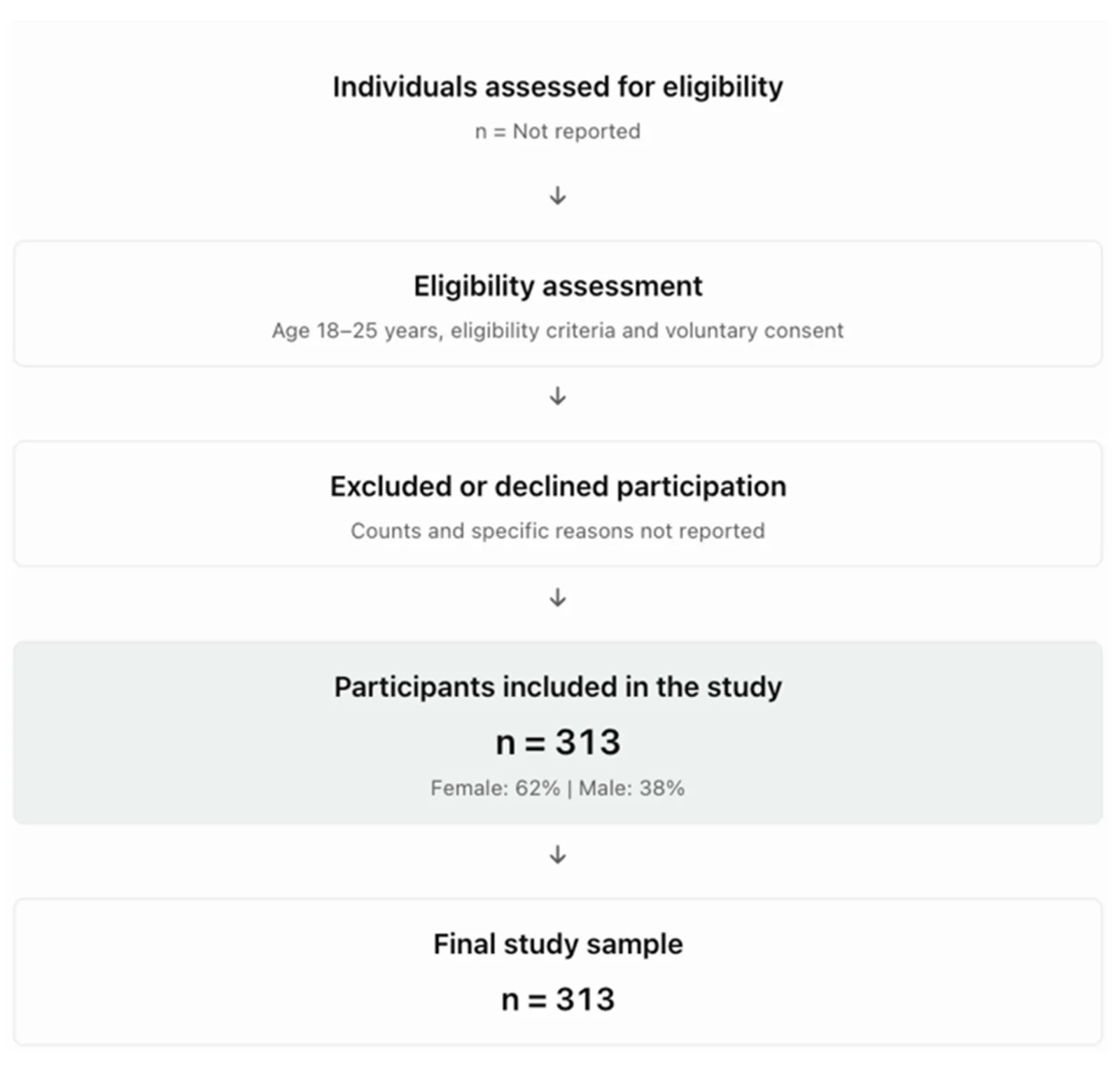
Flowchart of participant recruitment, eligibility assessment, and inclusion in the study.

Data collection was conducted through face-to-face interviews administered by the researchers in a quiet and appropriate environment. Before participation, individuals received information about the purpose and procedures of the study, and written informed consent was obtained. Completion of the data collection instruments required approximately 10–15 min per participant. Participants were given sufficient time to complete the questionnaires independently, without guidance or interference from the researchers. Following data collection, all responses were transferred to an electronic database and checked for missing values and data entry errors before statistical analysis. The participant recruitment, eligibility assessment, and inclusion process is illustrated in Figure 1.

### 2.2. Data Analysis

Statistical analyses were performed using IBM SPSS Statistics Windows, version 22.0 [20]. Descriptive statistics, including frequencies, percentages, means, and standard deviations, were used to summarize participants’ sociodemographic characteristics, HPV-related knowledge and vaccination awareness, as well as scores obtained from the HPV Awareness and Concern Level Scale, the HPV Testing Attitudes and Beliefs Scale, and the Health Belief Model Scale for HPV Infection and Vaccination.

Before conducting parametric analyses, the distributions of the scale scores were evaluated using skewness and kurtosis coefficients. Values within the commonly accepted ranges of ±1.5 and ±2.0 were considered indicative of approximate normality, in accordance with the relevant statistical literature [21,22]. Based on these assessments, the distributions were considered sufficiently close to normal to proceed with parametric analyses.

Independent-samples *t*-tests were used to compare scale scores between two independent groups, including comparisons based on gender, educational attainment, sexual intercourse experience, and contraceptive use. One-way analysis of variance (ANOVA) was used to examine differences in scale scores across categorical variables with more than two groups, including selected sociodemographic characteristics and HPV-related knowledge, screening practices, and vaccination perceptions. When statistically significant differences were identified through ANOVA, post hoc comparisons were conducted using Tukey’s honestly significant difference test or the least significant difference test to identify the groups responsible for these differences.

Pearson product–moment correlation analysis was performed to examine the direction, strength, and statistical significance of the relationships among total HPV awareness scores, total HPV testing attitudes and beliefs scores, and overall HPV infection- and vaccination-related health belief scores. All statistical tests were evaluated using a two-sided significance level of *p* < 0.05.

## 3. Results

An analysis of the descriptive characteristics of the participants revealed that 62% were female and 38% were male. The mean age of the participants was 22.03 ± 2.156 years, with an age range of 18–25 years. In terms of education level, 77.3% of the participants were university graduates and 22.7% were high school graduates. In terms of income, 76.4% were in the middle-income group, and 88.2% were single. In terms of sexual intercourse status, 35.5% of the participants reported having had sexual intercourse before, whereas 64.5% reported having no sexual experience. With respect to contraceptive use, 30.7% of the participants reported using some form of contraception, whereas 69.3% reported not using any. The most frequently preferred method of contraception was condoms with a rate of 81.2%, followed by oral contraceptives with 8.3% and withdrawal with 7.3%.

When examining Pap smear testing status, 54.3% of the participants stated that they had not had the test because they were not sexually active, 6.4% stated that they had it regularly, and 39.3% stated that they had never had a Pap smear. When opinions on the benefits of the Pap smear were evaluated, 21.4% of the participants stated that the test would be useful in the early diagnosis of cervical cancer. When their level of knowledge regarding the frequency of the test was examined, it was determined that 56.9% of the participants were not informed about this issue; 25.9% believed the test should be performed every three years, 11.2% every year, and 6.1% every five years.

When opinions on the relationship between HPV and cervical cancer were examined, 81.5% of the participants stated that there is a link between the viruses and cancer, while 18.5% stated that there is no such link. While 16.6% reported receiving education from their families about sexually transmitted infections, 83.4% reported receiving no training from their families. A total of 72.8% reported receiving information about HPV from outside their families, and 27.2% stated that they were not informed. When asked whether HPV causes cervical cancer, 50.5% of the participants believed that HPV causes cancer, 40.9% were undecided, and 8.6% stated that such a link was not found. Additionally, 75.4% believed that HPV can exist without symptoms, and 24.6% believed that the virus always causes symptoms. The most well-known HPV symptoms were warts in the genital area, with 88.2%, followed by warts in the penis and anus area, with 73.2%, and warts in the mouth and throat area, with 38.3%.

When the level of awareness about the HPV vaccine was examined, 86.3% of the participants reported having heard of the vaccine before, whereas 13.7% reported not having heard of it. Among the vaccine information sources, social media was the top source (66.5%), followed by other sources (21.1%) and medical advice (12.5%). Sixty-nine percent of the participants believed that individuals who were not sexually active should also receive the HPV vaccine, while 31% stated that the vaccine was not necessary until they had resumed sexual activity. The most common view on who should receive the HPV vaccine was that everyone should receive it (49.8%), followed by the view that only women should receive it (43.1%).

In an assessment of the age at which the HPV vaccine should be administered, 48.6% of participants indicated that the vaccine should be administered at age 18, 24.6% at age 9, 22.4% at age 15, and 4.5% at age 30. When the level of knowledge regarding the vaccine’s price was examined, it was determined that 63.9% of the participants were unaware of the price, whereas 31.3% knew that the vaccine cost between 3000 and 7000 TL. While 58.5% of the participants believed that the HPV vaccine should be administered to protect against cervical cancer, 38.3% were uninformed about the vaccine, and 3.2% stated that the vaccine is harmful and unsafe. When the participants’ families’ attitudes towards the HPV vaccine were examined, 51.4% stated that their families had different opinions, 25.9% stated that their families might perceive their vaccination as having sexual intercourse, and 22.7% stated that their families associated it with the possibility of contracting a sexually transmitted disease.

As shown in Table 1, the average total HPV awareness score average was calculated as 48.6 ± 11.2 (Min = 0, Max = 76). For the HPV awareness sub-dimensions, the average of the health sub-dimension was 23.1 ± 6.1, the average of the exclusion sub-dimension was 10.23 ± 4.51, and the average of the awareness sub-dimension was 15.3 ± 3.9. When the attitude and belief scale about HPV testing was examined, the total score average was found to be 80.2 ± 16.7 (Min = 20, Max = 123). In terms of sub-dimensions, the average of personal barriers was 22.4 ± 8.1, the average of social norms score was 14.8 ± 5.9, the average of confidence score was 30.8 ± 6.8, and the average of anxious attitude score was 12.3 ± 4.9. The overall mean of the HPV infection and vaccination health belief model scale was calculated as 2.6 ± 0.5 (Min = 1, Max = 4). For the sub-dimensions, the mean perceived severity was 2.98 ± 0.73, the mean perceived obstacle score was 2.1 ± 0.5, and the mean perceived benefit score was 2.9 ± 0.7.

According to the analysis findings in Table 2, a positive and significant correlation is found between the total HPV awareness score and the total HPV test attitude and belief score (r = 0.239; *p* < 0.01). This suggests that as HPV awareness increases, attitudes and beliefs about HPV testing also change in a positive direction. Furthermore, a significant and positive correlation is found between the total HPV awareness score and the overall HPV infection and vaccination health belief model score (r = 0.337; *p* < 0.01). This result suggests that individuals with high level of HPV awareness also have stronger health beliefs about HPV infection and vaccination. Similarly, a significant and positive correlation is found between the total HPV test attitude and belief score and the overall HPV infection and vaccination health belief model score (r = 0.228; *p* < 0.01). This finding suggests that positive attitudes and beliefs about HPV testing are linked to health beliefs about HPV vaccination.

According to the analysis results based on gender, the HPV awareness score (50.03 ± 10.51) was found to be statistically significantly higher in female participants compared to male participants (46.2 ± 11.9) (*p* = 0.004). Similarly, the HPV infection and vaccination health belief model score was also significantly higher in females than in males (*p* = 0.000). However, no statistically significant difference was found between genders in terms of HPV testing attitudes and belief scores (*p* = 0.069).

In the evaluation based on the education level variable, the HPV infection and vaccination health belief model score was found to be significantly higher in university graduates (2.66 ± 0.43) than in high school graduates (2.49 ± 0.54) (*p* = 0.018). However, no significant difference was found in the HPV awareness score or HPV testing attitude and belief scores based on education level (*p* > 0.05). Comparisons made by marital status, occupation, and income level revealed no significant differences in all scale scores (*p* > 0.05). Similarly, analyses conducted by sexual intercourse status and contraceptive use revealed no statistically significant differences between the groups (*p* > 0.05).

A statistically significant difference was observed in HPV infection- and vaccination-related health belief scores according to participants’ reported Pap smear testing status (*p* = 0.043). Post hoc analysis indicated that participants who reported not undergoing Pap smear testing because they were not sexually active had significantly higher health belief scores than those who reported never having undergone a Pap smear test for other or unspecified reasons. This finding reflects differences in HPV-related health beliefs between the reported response categories and should not be interpreted as evidence that sexual inactivity itself is associated with stronger health beliefs.

When opinions regarding the benefits of the Pap smear test were examined, a significant difference was found in terms of HPV infection and vaccination health belief model scores (*p* = 0.012). Post-hoc analysis revealed that participants who believed the test would be beneficial in early diagnosis of cervical cancer had significantly higher health belief model scores than participants with other views. Furthermore, a significant difference was found in terms of opinions regarding Pap smear frequency between HPV infection and vaccination health belief model scores (*p* = 0.001). Post-hoc analysis revealed that participants who believed the test should be performed every year or every three years had significantly higher scores than those who were unaware of the test.

When examining views on the virus–cancer relationship, statistically significant differences were found in terms of HPV awareness scores (*p* = 0.001) and HPV infection and vaccination health belief model scores (*p* = 0.002). Participants who believed that viruses caused cancer had significantly higher awareness and health belief scores compared to participants who held the opposite view.

An analysis based on whether they received education about sexually transmitted infections from their families revealed a significant difference in HPV infection and vaccination health belief model scores (*p* = 0.004). Accordingly, participants who received education on this topic from their families had higher health belief scores than those who did not.

An assessment based on whether they received information about HPV from outside their families revealed significant differences in HPV awareness (*p* = 0.000) and HPV infection and vaccination health belief model (*p* = 0.000) scores. Participants who reported receiving information from outside their families had statistically significantly higher awareness and health belief scores than those who were not informed.

When opinions on whether HPV causes cervical cancer were evaluated, statistically significant differences were found in HPV awareness (*p* = 0.000) and HPV infection and vaccination health belief model (*p* = 0.000) scores. According to the analysis results, participants who believed HPV caused cervical cancer had significantly higher scores compared to the other two groups.

A significant difference was found in HPV awareness (*p* = 0.000) and HPV infection and vaccination health belief model (*p* = 0.002) scores based on the belief that HPV can be present without symptoms. Accordingly, participants who believed HPV could be present without symptoms had significantly higher scores than those who believed the virus always showed symptoms.

An analysis based on knowledge about the HPV vaccine revealed significant differences in HPV awareness (*p* = 0.000) and HPV infection and vaccination health belief model (*p* = 0.001) scores. Participants who had heard of the HPV vaccine had significantly higher scores than those who had not.

## 4. Discussion

When the Pap smear status of the participants was examined, it was determined that the majority were not sexually active, and among those who were sexually active, only a small portion had ever had a Pap smear. It was also determined that a large portion of participants were unaware of the frequency of the test, and the percentage of those who believed the test was practical for the early diagnosis of cervical cancer was low. The literature reports that, despite being aware of cervical cancer screening, the number of women who had a Pap smear test and attended regular screenings was low [3]. This is believed to be due to a lack of education about the necessity of regular Pap smear testing. To address this educational gap, it is crucial for healthcare professionals to organize informative training programs for women. By increasing awareness of the importance of Pap smears and cervical cancer screening, healthcare professionals can encourage women to participate in regular screenings. This can increase the effectiveness of early diagnosis and preventive healthcare services.

Analyses of the relationship between HPV and cervical cancer revealed that most participants believed that there was a link between the virus and cancer. However, when their knowledge of sexually transmitted diseases was examined, it was observed that a significant portion of participants did not receive any training on the subject from their families. A study in the literature found that the information participants receive about sexually transmitted diseases came from healthcare personnel or teachers [23]. This result supports our study and is believed to be due to the taboo surrounding sexuality within traditional Turkish society, which prevents discussion among family members.

The study results revealed that a high percentage of participants believed that HPV causes cervical cancer, whereas a similar percentage of participants were undecided. The most recognized HPV symptom is genital warts, whereas the least known is warts in the mouth and throat. A study by Ajibola et al. [24] revealed that the association between HPV and cervical cancer is widely known, but the percentage of participants aware of its connection to throat, anal, and penile cancers is low. The results of our study are consistent with these findings. Furthermore, the lack of sufficient knowledge among participants about HPV symptoms highlights the need for effective educational programs. Therefore, it is important to increase awareness and training programs aimed at raising awareness.

In our study, participants were found to have heard of the HPV vaccine and mostly learned about it from social media. A separate literature review revealed that more than half of the participants had not heard of the HPV vaccine, and that the majority of those who had heard about it obtained this knowledge from a healthcare professional [25]. The fact that healthcare professionals were not the preferred source of information about HPV and the HPV vaccine in our study demonstrates a lack of knowledge among healthcare professionals in this area. Compared with Demir’s study [25], it was also observed that HPV awareness levels increased due to the influence of social media.

In our study, most participants knew that sexual activity was not required for HPV vaccination and believed that everyone should receive the vaccine. A study by Boakye et al. [26] revealed that sexually active individuals had higher rates of HPV vaccination than did inactive individuals. A study in the literature also revealed that most participants believed that the HPV vaccine would be more effective if administered between the ages of 9 and 14 [27]. The participants in our study understood that they had misconceptions about the timing of HPV vaccination. However, they were correctly informed that there was no link between sexual intercourse and vaccination. While the prevailing view in our study was that everyone, male or female, should receive the HPV vaccine, a study conducted with male healthcare workers in the literature revealed that the view was that only women should receive the HPV vaccine [28]. This inaccurate view among male healthcare workers is an indicator of gender inequality and an obstacle to the widespread adoption of the HPV vaccine in society.

While our study revealed a lack of information about the cost of the HPV vaccine, a study in the literature revealed that the high cost of the HPV vaccine is a significant factor influencing willingness to receive the vaccine [29]. Individuals who are knowledgeable about the HPV vaccine and want to be vaccinated may be unable to obtain it due to financial constraints. Most participants in our study were aware that the HPV vaccine is a protective factor against cervical cancer. The study by Jorgensen et al. [30] also supported this view, finding that its benefits outweigh its harms. However, it was also emphasized that the risks are not adequately reported, and therefore the balance between benefits and harm is unclear. While our study concluded that the families of participants had varying opinions about HPV vaccination, the literature indicates that the vast majority have no opinion about the HPV vaccine [31]. Once again, a lack of knowledge negatively impacts HPV vaccine acceptance.

The high total HPV awareness score in the present study indicates a relatively high level of awareness of HPV. The lower levels of the health and exclusion subscales indicate that individuals, despite having knowledge, experience inadequate preventive health care. The relationship between the levels of conscious awareness levels and HPV knowledge levels of midwifery and nursing students have been examined in the literature. These findings indicate that students’ knowledge about HPV is inadequate, and that their conscious awareness is low. [32] Compared with studies in the literature, the inadequacy of individuals’ HPV preventive health care highlights the need for effective health education and information activities throughout society. This observed lack of awareness not only makes preventing HPV-related diseases challenging but also leads to the inadequate adoption of preventive health services such as vaccination.

When the subscales of the HPV Testing Attitudes and Beliefs Scale were examined, trust emerged as an important dimension of participants’ attitudes toward HPV testing, alongside personal barriers. In this context, trust refers to confidence associated with HPV testing and the testing process rather than confidence in vaccine effectiveness or the scientific literature. Similarly, Cangöl et al. [18] reported that women experienced personal and social barriers to HPV testing and identified trust as an important component of testing-related attitudes. These findings highlight the importance of providing clear and accurate information about HPV testing procedures and addressing individuals’ concerns to support informed screening decisions. Furthermore, the present study revealed that perceived seriousness had the highest mean score among the HPV infection- and vaccination-related health belief dimensions, whereas perceived barriers had the lowest mean score. Similarly, Sezgin et al. [33] emphasized the importance of perceived seriousness and barriers in HPV-related health beliefs. These findings suggest that recognizing HPV infection as a serious health concern does not necessarily eliminate perceived barriers to vaccination, highlighting the need for educational interventions that address both risk perceptions and vaccination-related concerns.

The results of the analysis conducted to examine the relationship between the scores on the scales used in the study indicate that increased HPV awareness positively affects attitudes and beliefs about HPV testing. The results of a study in the literature are consistent with those of our study [34]. Our study and other studies in the literature indicate that individuals with high levels of HPV awareness also have stronger health beliefs about HPV vaccination [11]. A study conducted by Akpınar and Tosun with university students revealed that belief in HPV testing is positively correlated with health beliefs about HPV vaccination [29]. These findings support our study.

When the gender analysis of the participants in our study was examined, it was found that women had significantly higher HPV awareness and HPV infection and vaccination health belief model scores than men. Various studies in the literature also support our results [35,36].

This study revealed that health belief model scores for HPV infection and vaccination differ significantly because of regular Pap smear testing. These findings suggest that participants who are not sexually active may have greater awareness of the HPV vaccine and more positive beliefs about getting it. The literature also emphasizes that knowledge increases preventive health behaviors [37,38]. Among sexually active individuals, the low rates of Pap smear testing may be due to lack of knowledge, embarrassment, and misconceptions. In our study, most participants agreed that the Pap smear test is an early diagnostic method for cervical cancer, demonstrating a high level of knowledge and awareness of this issue. Demir’s [25] study revealed that the participants’ awareness levels regarding HPV infection and the HPV vaccine were low and that they lacked knowledge about the Pap smear test, an early diagnosis method. As a result of this lack of knowledge, the majority did not undergo Pap smear testing or receive the HPV vaccine.

The significantly higher scores of individuals who considered regular HPV testing than those who were unaware of HPV indicated that awareness levels positively impact preventive health behaviors. The literature indicates that the average HPV knowledge score (2.73 ± 2.46) of 546 female students studying at a Vocational School was low; moreover, among the 31.5% of those who knew that the HPV vaccine was protective against cervical cancer, only 2.9% were vaccinated. [33] Compared with Sezgin’s study, both studies demonstrated that a lack of knowledge about the HPV vaccine [33] significantly limits the use of preventive health services; moreover, awareness of HPV strengthens preventive health services.

In this study, participants who believed that HPV could cause cancer obtained significantly higher scores on the HPV Awareness Scale and the Health Belief Model Scale for HPV Infection and Vaccination than those who did not share this belief. Similarly, a previous study conducted among Faculty of Health Sciences students reported an association between awareness of HPV-related cancer risks and HPV-related health belief scores [39]. These findings suggest that recognizing the relationship between HPV infection and cancer may be associated with individuals’ perceptions of HPV-related health risks and vaccination. However, higher scores on health belief measures should not be interpreted as direct evidence of greater engagement in preventive health behaviours. Therefore, educational interventions should aim to improve awareness of HPV-related cancer risks while addressing specific perceptions and concerns regarding vaccination and screening.

The study revealed that individuals who received HPV education from their families had greater health beliefs, indicating that early family education increases their propensity for health protection. A study by Sezgin et al. [33] concluded that parental education level had a significant effect on students’ HPV knowledge. These findings suggest that family education positively impacts individuals’ knowledge and health beliefs about HPV. Both studies demonstrated that the STD education received from families positively influences health attitudes about HPV and is a determinant of health-related decisions [33]. The study revealed that individuals who received information about HPV from outside their families had more awareness and stronger health perceptions than those who did not. They were observed to engage in more positive health behaviors for reasons such as the scientific and clear nature of the information. In the literature, a study conducted with university students revealed that students had higher levels of knowledge about HPV and the vaccine, which was reflected in their health belief model scores. After the training, 42.6% of the unvaccinated participants stated that they wanted to be vaccinated, demonstrating that the information had an impact on their health beliefs [40]. Both studies revealed that health education positively impacts HPV and susceptibility to infection, leading to more informed decisions about vaccination. Disseminating HPV education programs to a wider audience is crucial for increasing HPV health belief perceptions and awareness of vaccination.

In our study, differences were found between the HPV awareness, HPV infection, and vaccination health belief model scores. The belief that HPV would not cause symptoms was high, highlighting a lack of knowledge. Similarly, in the study by Akpınar and Tosun [29], most participants were unaware that HPV infection could be present in the body without symptoms. HPV infection can often be asymptomatic, and participants in our study having this knowledge could be protective for their own health.

## 5. Conclusions

This study revealed that, although young adults demonstrate relatively high awareness regarding the human papillomavirus (HPV) and its link to cervical cancer, significant knowledge gaps and misconceptions persist concerning the appropriate timing, target audience, and protective benefits of the HPV vaccine. The fact that social media is most frequently cited as the source of information regarding the vaccine underscores the importance of reliable digital health communication. Consistent with the research hypothesis, significant positive correlations were found between HPV awareness and attitudes/beliefs regarding HPV testing (r = 0.239, *p* < 0.01), as well as between HPV awareness and health beliefs regarding vaccination (r = 0.337, *p* < 0.01). Furthermore, female participants exhibited higher scores for HPV awareness and vaccination-related health beliefs compared to male participants. These findings highlight the need for targeted educational interventions, gender-inclusive awareness programs, evidence-based social media campaigns, and counseling services provided by health professionals to dispel misconceptions and strengthen preventive health knowledge regarding HPV. Prospective (longitudinal) studies and intervention trials are recommended to determine whether increased awareness translates into higher vaccination rates and improved preventive health behaviors.

## 6. Contributions to the Literature

- This study contributes to the limited evidence on HPV vaccine awareness and health beliefs among young adults in Turkey.
- The findings highlight persistent misconceptions about the timing, accessibility, and cancer-related risks of the HPV vaccine, other than cervical cancer.
- The study demonstrates the significant role of social media and informal information sources in shaping knowledge and attitudes regarding HPV.
- The results highlight the need for population-based education strategies conducted by health professionals to improve vaccine acceptance and preventive health behaviours.
- The study supports the importance of strengthening public health policies for HPV vaccine and awareness programs among young adults.

## Figures and Tables

**Table 1:** HPV Awareness, HPV Test Attitudes and Beliefs, and HPV Infection Vaccination Health Belief Model Mean Scores.

	N	Average	SD	Min.	Max.	Kurtosis	Skewness	Alpha
**HPV Awareness Total**	313	48.6	11.2	0.0	76.0	0.7	−1.0	0.9
Health	313	23.1	6.1	0.0	32.0	1283.0	−0.8	0.9
Exclusion	313	10.2	4.5	0.0	20.0	−0.2	−0.1	0.9
Awareness	313	15.3	3.9	0.0	24.0	1.0	−0.5	0.9
**HPV Testing Attitudes and Beliefs Total**	313	80.2	16.7	20.0	123.0	1335.0	−0.6	0.9
Personal Barriers	313	22.4	8.1	7.0	46.0	−0.5	0.2	0.9
Social Norms	313	14.8	5.9	4.0	28.0	−0.7	−0.2	0.9
Trust	313	30.8	6.8	6.0	41.0	2033.0	−1322.0	0.8
Anxiety Attitude	313	12.3	4.9	3.0	21.0	−0.6	−0.2	0.8
**HPV Infection Vaccination Health Belief Model General**	313	2.6	0.5	1.0	4.0	1235.0	−0.8	0.9
Perception of Seriousness	313	3.0	0.7	1.0	4.0	0.0	−0.7	0.9
Perception of Barriers	313	2.1	0.6	1.0	4.0	0.7	0.5	0.9
Perception of Benefits	313	2.9	0.7	1.0	4.0	0.1	−0.4	0.9

**Table 2:** Correlation Analysis of HPV Awareness, HPV Testing Attitudes and Beliefs, and HPV Infection Vaccination Health Belief Model Scores.

		HPV Awareness Total	HPV Testing Attitudes and Beliefs Total	HPV Infection Vaccination Health Belief Model General
HPV Awareness Total	r	1.000		
	*p*	0.000		
HPV Testing Attitudes and Beliefs Total	r	0.239 **	1.000	
	*p*	0.000	0.000	
HPV Infection Vaccination Health Belief Model General	r	0.337 **	0.228 **	1.000
	*p*	0.000	0.000	0.000

** <0.01; Pearson Correlation Analysis.

## Data Availability

All relevant data are within the manuscript. Any further requests regarding, the data used for this study can be made through the corresponding author.
